# Application of electronic nose technology in the diagnosis of gastrointestinal diseases: a review

**DOI:** 10.1007/s00432-024-05925-w

**Published:** 2024-08-27

**Authors:** Tan-tan Ma, Zhiyong Chang, Nan Zhang, Hong Xu

**Affiliations:** 1 Department of Gastroenterology, The First Hospital of Jilin University, 71 Xinmin Street, Changchun, 130021 China; 2 Key Laboratory of Bionic Engineering, Ministry of Education, Jilin University, Changchun, 130022 China

**Keywords:** Electronic nose technology, Gastric cancer, Colorectal cancer, Barrett’s esophagus, Inflammatory bowel disease

## Abstract

Electronic noses (eNoses) are electronic bionic olfactory systems that use sensor arrays to produce response patterns to different odors, thereby enabling the identification of various scents. Gastrointestinal diseases have a high incidence rate and occur in 9 out of 10 people in China. Gastrointestinal diseases are characterized by a long course of symptoms and are associated with treatment difficulties and recurrence. This review offers a comprehensive overview of volatile organic compounds, with a specific emphasis on those detected via the eNose system. Furthermore, this review describes the application of bionic eNose technology in the diagnosis and screening of gastrointestinal diseases based on recent local and international research progress and advancements. Moreover, the prospects of bionic eNose technology in the field of gastrointestinal disease diagnostics are discussed.

## Background

Gastrointestinal diseases, including functional and organic diseases of the esophagus, stomach, small intestine, and large intestine, are encountered frequently in clinical practice. The clinical manifestations of gastrointestinal diseases include symptoms associated with digestive dysfunction and are often accompanied by clinical manifestations of other systems. According to recent epidemiological surveys, the incidence and prevalence of gastrointestinal disorders are rising, exerting a substantial negative effect on patients' health, and incurring considerable social and psychological costs. A study covering 204 countries and regions reported that the age-standardized incidence of digestive diseases worldwide was 95,582 cases per 100,000 individuals in 2019, accounting for more than one-third of epidemic disease cases (Wang et al. 2023).Therefore, early diagnosis of gastrointestinal diseases is crucial for timely treatment. Currently, routine clinical examination methods for detecting gastrointestinal diseases include complete blood cell counts, blood urea nitrogen levels, tumor markers, and C-reactive protein levels; abdominal computed tomography; and endoscopy. However, conventional diagnostic methods have limitations. First, gastrointestinal disorders are associated with a wide range of symptoms, and a definitive diagnosis can only be established through examination and laboratory tests. In most basic hospitals, access may not be universal or timely due to a variety of examination methods, need for trained personnel, and instrumental analysis. Second, intraprocedural patient intolerance and financial expenses also limit the broad application of endoscopy. Gastrointestinal diseases are chronic, and repeated examinations place medical and economic burdens on patients. Therefore, there is a demand for economical, less invasive, and simpler diagnostic interventions in clinical practice to meet the patient’s medical needs and reduce their economic burden. Owing to scientific and technological advancements, technologies related to the discovery of volatile organic compounds (VOCs) and their role in diagnostics have garnered considerable attention (Scheepers et al. 2022).

**Fig. 1:**
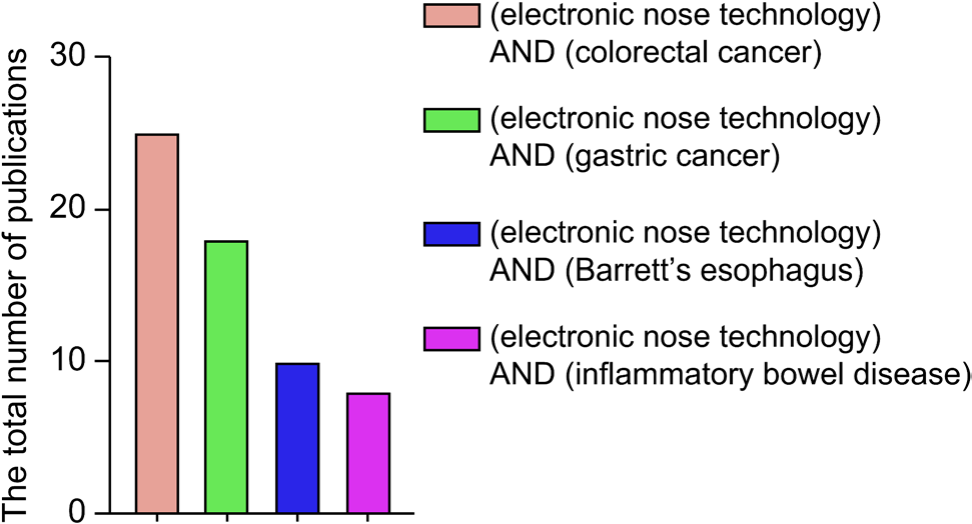
The total number of publications over the last 15 years on the subject using different keywords

Electronic nose (eNose) technology has found increasing application in medical diagnosis, especially for gastrointestinal diseases. We conducted a systematic literature review to comprehensively evaluate the latest advances and results of research investigating the role of eNoses in the diagnosis of gastrointestinal diseases. We used rigorous inclusion and exclusion criteria to ensure that the included studies were directly relevant and of reliable quality. Specifically, we conducted a comprehensive search of authoritative databases such as PubMed, Web of Science, Scopus, etc., using the following search terms: “electronic nose,” “e-nose,” “gas sensor array,” “gastric cancer,” “colorectal cancer,” “Barrett’s esophagus,” and “inflammatory bowel disease.” The search time frame covers nearly fifteen years to capture the latest research findings. Through a rigorous selection process, we identified several high-quality research articles, which provided a solid literature foundation for the review of the diagnostic role of eNose in gastrointestinal diseases (Fig. 1).

## Generation of VOCs

The discovery of VOCs signals a new frontier in medical diagnostics because of their noninvasive and inexpensive nature (Broza and Haick 2013). VOCs are generated by metabolic processes during the cell’s life cycle. They are mainly composed of hydrocarbons, oxyhydrocarbons, halogenated hydrocarbons, nitrohydrocarbons, and sulfur-containing hydrocarbons. They also have high vapor pressures and low boiling temperatures. Owing to their low molecular weight, small size, and volatility, these compounds spread through blood circulation to distant organs and can be excreted or secreted in various body fluids. Low concentrations of VOCs are detected in peripheral blood, urine, sweat, feces, and exhaled gases. Therefore, human physiological metabolism produces numerous VOCs in its secretions and excretions. Since VOCs are the endogenous products of normal cells and microorganisms, they can provide qualitative information related to an individual’s metabolism (Buszewski et al. 2007; Hakim et al. 2012; Broza and Haick 2013; Nakhleh et al. 2014; Broza et al. 2018). Consequently, VOCs reflect cellular metabolic changes and pathophysiological processes in humans. Moreover, VOCs are easily sampled and can be used for the non-traumatic measurement of diagnostic biomarkers and detect disease activity. VOCs emitted from various parts of human organs vary according to the age, diet, sex, and physiological status. Therefore, VOCs can be considered the “odor fingerprint” of an individual.

Cells respond to systemic or local stimuli by activating various relevant signaling pathways and cascade reactions. These fast metabolic processes alter cellular basal activities or features, allowing them to adapt to the constantly changing needs of the microenvironment. In the diseased state, the human body produces a series of pathophysiological responses, leading to altered metabolic levels (Haick et al. 2014). These bodily changes occur at the single cellular level or throughout an entire organ, e.g., the lung, liver, or kidney, to preserve the internal environment. VOCs can reflect various metabolic changes such as inflammation, necrosis, cancer, and alterations in the microbiome. Moreover, VOCs are associated with external factors, such as environmental pollutants, drugs, and diet.

In 1971, Linus Pauling discovered hundreds of VOCs in exhaled gases and urine, laying the foundation for research on VOCs in the human body (Pauling et al. 1971). Exhaled-gas analysis is a new research field with a long history. Researchers started studying the relationship between the odors of exhaled gases and diseases as early as 460 to 370 BC. According to biologists, odors exhaled by the human body consist of hundreds of gaseous compounds (Amann et al. 2014). These compounds have extremely high inter-individual variability, and the concentration of each compound depends on various elements, including metabolic and pulmonary or physiological differences. However, these compounds have potential value for medical diagnosis, therapeutic monitoring, disease status assessment, and investigation of physiological and pathophysiological conditions. Therefore, the odors found in exhaled gases are closely related to the body’s overall health (Pizzini et al. 2018; Ruszkiewicz et al. 2020).

The human body does not emit abnormal odors in a healthy state; however, in a diseased state, distinct abnormal odors are emitted through the skin mucosa, respiratory secretions, gastrointestinal secretions/excretions. These abnormal odors may indicate specific symptoms of certain diseases. For example, patients with diabetes tend to emit a pyruvic acid odor, the breath of patients with typhoid often smells like “baked bread,” the breath of patients with tuberculous lymphadenitis emits a beer-like odor, a chicken feather odor is primarily associated with rubella, a musty odor is mainly found in patients with liver diseases, and those with lung ulcers or bronchial dilatation with infection often have fetid breath. Currently, detection methods for volatile metabolites (which function as biomarkers) include bionic eNose technology, proton-transfer-reaction mass spectrometry, ion flow tube mass spectrometry, and gas chromatography-mass spectrometry.

## Overview of the eNose system

**Fig. 2:**
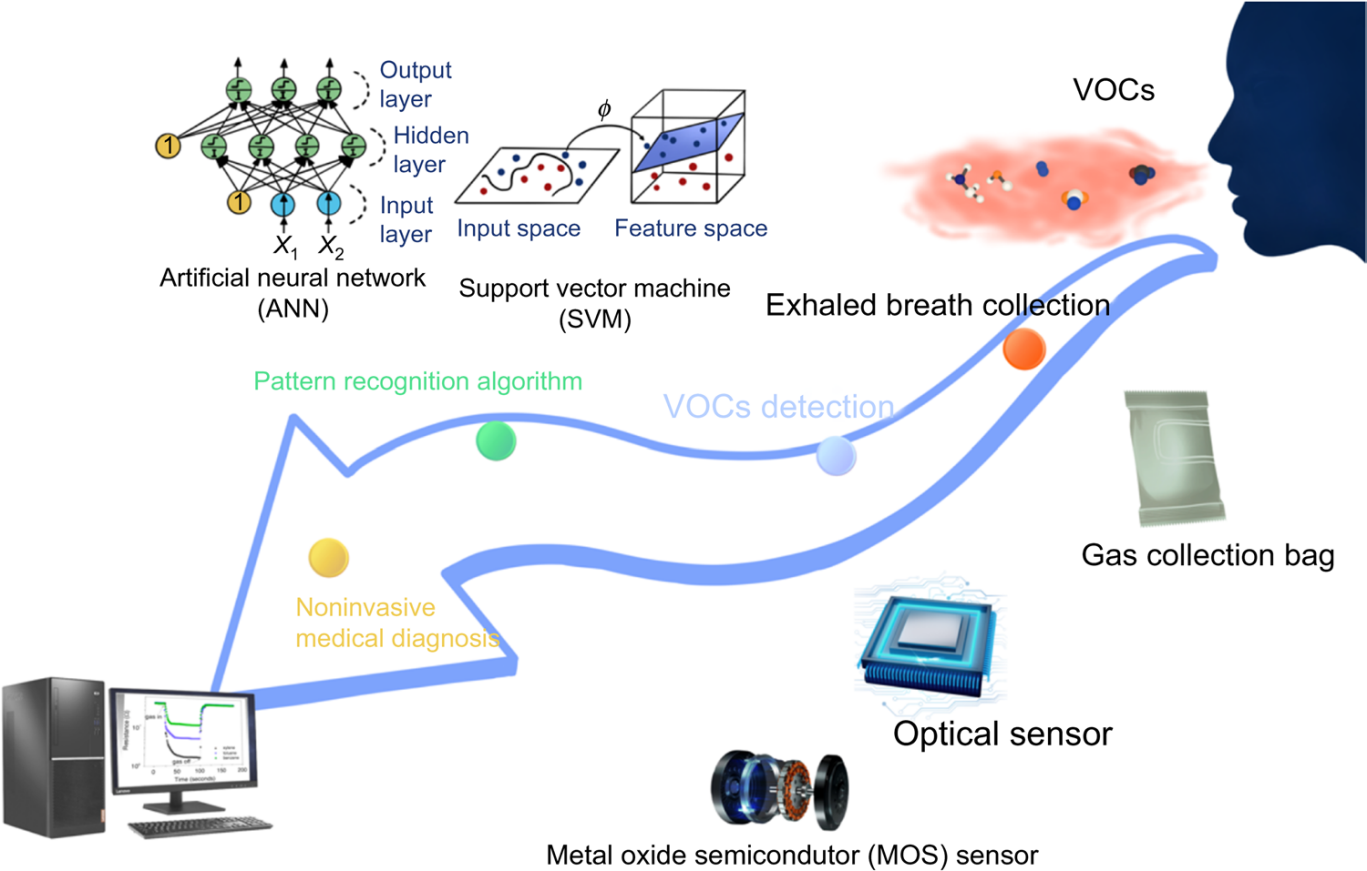
Schematic diagram of the noninvasive breath detection by the eNose system

An eNose is an electronic nose system that uses the response patterns of a gas-sensor array to identify odors. In 1964, Hatman and Wilkens used the oxidation–reduction reaction of gases on electrodes to electronically simulate the olfactory process. In 1982, Persaud and Dodd (Persaud and Dodd 1982) were the first to propose the use of eNose as an intelligent chemical sensor array for gas classification. They detected different volatile odors by simulating the human olfactory system at different stages. Research groups first proposed the concept of pattern recognition at the 8th Annual Meeting of the European Research Organization for Chemical Sensors in 1987. More advanced eNose technologies have been gradually explored and developed, boosting widespread academic interest. Inspired by the olfactory system, researchers combined a chemical sensor array with pattern recognition technology in an artificial device (i.e., an eNose) that can serve as a cost-effective chemical detector. In 1989, eNose was first defined at the Chemical Sensors and Information Processing Conference as “a device that can identify single and complex odors and consists of multiple gas-sensitive sensors with overlapping performance and an appropriate pattern classification method” (Gardner and Bartlett 1994). Subsequently, the first International Conference on eNoses was held in Iceland in 1990, and since then, research on eNose technology has advanced significantly (Fig. 2).

The sense of smell is produced by the human olfactory system working in concert with other organs, especially the brain. Researchers who elucidated the functioning of the olfactory system were awarded the 2014 Nobel Prize in Physiology or Medicine (Buck 2004). After the molecules carrying specific odors activate the odor receptors (i.e., the binding proteins of the receptors on the cilia of the olfactory epithelia), the odor receptor cells produce electrical signals that are transmitted to the structure (i.e., the olfactory bulb) responsible for olfactory processing in the brain, which in turn conveys olfactory information to the olfactory cortex. The odor information is then transmitted to the brain’s higher-level cortical areas associated with identifying and discriminating odor information and to the limbic area that may regulate emotions and motivational responses. The signals are identified based on the attributes and concentration of the smell, and the information is combined into a specific pattern, resulting in olfactory perception. The human brain can recall different signals resulting from diverse odor molecules, enabling the identification of different odors.

eNose comprises three major systems, viz. the odor sensor array, data processing, and pattern recognition systems. The core component is a gas sensor array that mimics the mammalian sense of smell. Sensor technology has rapidly evolved over the past few decades, leading to the development of various sensor formats and sensors with complex microarrays. In eNose systems, various physicochemical technologies have been employed to produce sensor arrays for odor characterization (Turner and Magan 2004).

Many olfactory receptor cells in the olfactory system are analogous to a gas sensor array. Each sensor can simultaneously react with multiple VOCs, and multiple sensors can simultaneously respond to the same VOCs. However, all sensors have different sensitivities toward a mixture of VOCs, offering significant advantages in determining complex and changeable breath signals. When the sensors come into contact with VOCs, they convert the chemical signals on the surface of different VOC molecules into measurable electrical signals, and these specific respiratory signals (i.e., odor-corresponding patterns) can be measured and quantified (Leopold et al. 2015; V A et al. 2021). Ultimately, each sensor generates a response spectrum toward a mixture of VOCs, which constitutes the overall response profile of the sensor array toward this mixture. Each sensor possesses differential sensitivity toward the measured gases, and the response spectra of the entire sensor array vary for different VOC mixtures. The eNose system can perform pattern recognition for different mixtures of VOCs based on different response profiles generated by the sensor array. The response spectra generated by the sensors must undergo signal preprocessing. The signal preprocessing methods include differential, relative, logarithmic, sensor value normalization, and array normalization algorithms. The preprocessed signal is then processed using a data-processing analyzer, an intelligent interpreter, and a knowledge base. Data on the relationships between gases and signals obtained after training are stored in the knowledge base of the pattern recognition system. Consequently, the gas signals to be measured can be compared with those in the knowledge base, thereby achieving gas recognition. Currently, the pattern recognition methods commonly used to analyze these datasets are statistical pattern detection techniques, such as principal component analysis, hierarchical cluster analysis, linear discriminant analysis, support vector machines, and neural network modeling, including artificial and evolutionary neural network techniques.

The advantages of eNose technology include its noninvasiveness, rapid measurement, real-time analysis, simple operation, and low cost. A sensor-array system consists of various sensors that detect and identify substances and environmental features. Common types of sensor array systems are surface acoustic waves (used for gas and liquid detection) (Chen et al. 2005; Rauch et al. 2018), quartz crystal microbalances (used for gas and liquid detection) (Grate 2000; Öztürk et al. 2016), metal oxide semiconductors (frequently used for gas detection) (Yu et al. 1999; Gardner et al. 2000; Leunis et al. 2014), conductive polymers (used for the detection and quantification of gases and liquids) (Vaddiraju and Gleason 2010), and carbon nanofibers (used to detect the presence and concentration changes of gases and liquids) (Consales et al. 2006). Nevertheless, eNoses based on metal oxide semiconductors are the most widely used.

eNose technology has been applied in many fields, including environmental monitoring (Wilson 2012; He et al. 2017); food safety (Aleixandre et al. 2008; Khaled et al. 2021); the determination of dangerous substances such as explosives, toxic gases, and chemical leaks, detection of explosives or accelerants; pharmaceuticals (Montuschi et al. 2013; Li et al. 2015); biomedicine (Gardner et al. 2000; Wilson and Baietto 2011; Bruins et al. 2013; Kou et al. 2017; van de Goor et al. 2017; Behera et al. 2019); and several other areas in applied science. In April 2022, the Food and Drug Administration issued an Emergency Use Authorization for exhaled air to test for coronavirus disease (COVID-19) infection in an individual. This method mainly detects 5 VOCs in the exhaled breath associated with new COVID-19 infection. The technique uses the InspectIR COVID-19 Respiratory Detector, which utilizes gas chromatography–mass spectrometry to separate and identify chemical mixtures and rapidly detect VOCs associated with SARS-CoV-2 infection in exhaled breath.

eNose technology can detect chemical fingerprints to identify different diseases (McWilliams et al. 2015; Yan et al. 2015; Sanaeifar et al. 2017). In clinical diagnosis, eNoses can be used to detect several human diseases, facilitating early diagnosis and treatment. Thus, eNoses has significant clinical application prospects as a noninvasive screening modality for gastrointestinal diseases.

In this review, we summarize the development of eNose technology, its application in diagnosing gastrointestinal diseases, and developmental trends in its application.

## eNose in the diagnosis of gastrointestinal diseases

### eNose in the diagnosis of gastric cancer

Gastric cancer (GC), a common malignancy, is the fifth most prevalent cancer worldwide and constitutes the fourth leading cause of cancer-related deaths. Early diagnosis of GC is crucial for improving patient survival rates. However, some patients are asymptomatic in the early stage and often progress to advanced stages at the time of clinical diagnosis. In addition, imaging techniques used clinically for the screening and diagnosis of GC, such as barium meal and abdominal computed tomography, are beset by disadvantages such as high costs, radiation damage, and low sensitivities and specificities. Although endoscopy with pathological biopsy is the most reliable method for diagnosing GC and plays a crucial role in disease screening, its widespread use is limited by invasiveness and technical expertise requirements. Exhaled VOC analysis is a cost-effective and easy-to-operate noninvasive assay with no adverse physical effects on patients. Breath VOCs have been extensively studied nationally and internationally, revealing their potential as a detection method for GC (Polaka et al. 2022). Breathomics is a branch of metabolomics that functions as a diagnostic aid by identifying and quantifying specific VOCs or VOC patterns associated with various diseases or physiological conditions resulting from disease-induced changes in metabolic processes (Daniel and Thangavel 2016; Einoch Amor et al. 2019). Currently, breath analysis is successfully used to diagnose lung, breast, gastric, prostate, colorectal, ovarian, head and neck, kidney, and bladder cancers (Peng et al 2010; Yang et al. 2021). Sensor-based gas chromatography has found significant application prospects in scientific research and clinical practice for the early detection of GC because of its low cost, noninvasiveness, high accuracy, and ease of operation (Miekisch et al. 2004; Amal et al. 2014; Haddad et al. 2020; Zhang et al. 2021; Xiang et al. 2021; Gouzerh et al. 2022). The application of eNose technology can help in the early detection of pre-cancerous lesions and ultimately reduce GC mortality. eNose performance depends on its sensitivity, accuracy, specificity, and predictive values (Shreffler and Huecker 2020; Yang et al. 2021). With advancements in technology, breath analysis methods may become critical for the detection of GC, providing more intensive information on the progression, specific VOCs, origins, and biochemical mechanisms underlying GC. VOCs contain valuable information regarding biochemical metabolism in cancer (Van Der Schee et al. 2018). Some compounds are associated with specific cancers and can be used to distinguish patients from healthy individuals (Xiang et al. 2021). Aldehydes and ketones are slightly soluble in blood and can be identified in the breath a few minutes after their release from tissues (Haick et al. 2014).

Oxidative stress, a major source of non-branched hydrocarbons in the body, causes lipid peroxidation of polyunsaturated fatty acids in cell membranes, leading to the generation of saturated alkanes, C3-C11 hydrocarbons such as ethane and pentane (Okunieff et al. 2005; Vousden and Ryan 2009). These cells are affected by pre-cancerous lesions or cells elsewhere in the body due to systemic oxidative stress. Since different cell types have different cell membranes, the VOCs emitted also differ.

Schuermans et al. (2018) investigated a breath test based on a miniature metal oxide gas sensor on exhaled breath samples to distinguish between patients with GC and healthy individuals. They used discriminant factor analysis (DFA) pattern recognition to develop a prediction model. The baseline attributes differed significantly only by age, with a mean age of 37 and 57 years for the healthy and patient groups, respectively (*p* = 0.000). Weight loss was the only symptom that showed a significant difference (*p* = 0.040). The study included 16 patients and 28 controls, of whom 13 were true positives and 20 true negatives. The sensitivity, specificity, and accuracy of the receiver operating characteristic (ROC) curve were 81%, 71%, and 75%, respectively. The positive and negative predictive values were 62% and 87%, respectively. This preliminary study suggested that eNose can be used to diagnose GC based on exhaled gases, showing promise as a predictive tool for GC screening.

Xu et al. (2013) demonstrated that a nanomaterial-based sensor effectively distinguished between patients with GC (*n* = 37) and nonmalignant gastric disease (*n* = 93) by developing a DFA model and analyzing 130 breath samples. The sensitivity and specificity for differentiating between GC and benign gastric disease were 89% and 90%, respectively. The sensitivity and specificity for distinguishing early GC (stages I and II) from advanced GC (stages III and IV) were 89% and 94%, respectively. The sensitivity and specificity for distinguishing benign ulcers from less severe gastric diseases (including 32 cases without an anomaly on gastroscopy and 29 with anomalies on gastroscopy but without an ulcer) were 84% and 87%, respectively.

Amal et al. (2016) collected 968 breath samples from 484 patients (including 99 with GC) for two analyses. The first sample was analyzed using gas chromatography–mass spectrometry with a multiple-corrected *t*-test (*p* < 0.017), whereas the second one was subjected to a cross-reactive nanoarray combined with pattern recognition. For the latter, the randomly selected training set comprised 70% of the sample, and the remaining 30% formed the validation set. The presence or absence and the risk level of pre-cancerous lesions were stratified using the Operative Link on Gastric Intestinal Metaplasia (OLGIM) assessment staging system. Patients with OLGIM stages III and IV were considered be at high risk. Based on the gas chromatography–mass spectrometry results, patients with cancer and high-risk patients had a unique composition of breath fingerprints. Eight significant VOCs were detected in the exhaled gases (*p* = 0.017). In contrast, nanoarray analysis revealed that the sensitivity, specificity, and accuracy for identifying patients with GC and controls (OLGIM stages 0–IV) were 73%, 98%, and 92%, respectively. The sensitivity, specificity, and accuracy of classification were 97%, 84%, and 87%, respectively, when comparing GC with OLGIM stages 0–II, and 93%, 80%, and 90%, respectively, when comparing GC and OLGIM stages III–IV. However, the sensitivity, specificity, and accuracy for the combination of OLGIM stages I–II, III–IV, and heterogeneous hyperplasia were 83%, 60%, and 61%, respectively. Consequently, nanoarray analysis may serve as a noninvasive screening and monitoring technique for GC and relevant pre-cancerous lesions.

### eNoses in the diagnosis of Barrett’s esophagus

Barrett's esophagus (BE) is a precancerous esophageal lesion, with the potential to develop into an adenocarcinoma upon malignant transformation. Thus, awareness of this condition and its early detection, appropriate treatment, and follow-up should be more widespread. Screening and monitoring for BE are aimed at early detection and reducing the mortality rate of esophageal adenocarcinoma. BE is characterized by the replacement of squamous epithelia in the distal esophagus with metaplastic (intestinal-type) epithelia due to gastroesophageal reflux. Endoscopy with pathological biopsy is the gold standard for diagnosing and monitoring BE. The annual incidence rate of esophageal adenocarcinoma in patients with BE is 0.3–0.6%, and the prevalence rate is approximately 1–2% in the general population (Boeckxstaens et al. 2014; Hayeck et al. 2010; Dumoulin et al. 2022). As most patients with BE are asymptomatic, its true prevalence rate may be underestimated. Changes that occur during monitoring intervals depend on the extent of atypical hyperplasia, and endoscopic eradication therapy is limited to patients with BE and confirmed atypical hyperplasia. The current guidelines recommend endoscopic screening and monitoring based on various risk factors; however, these factors are limited by invasiveness, availability of experienced specialists, and the physical, psychological, and economic burden on the patient. Transnasal endoscopy is a less invasive approach with similar limitations, such as the need for trained specialists and high costs.

In contrast, non-endoscopic methods require minimal intervention, can be performed in the consultation room, and are potentially a more desirable option for large-scale public screening and monitoring. The analysis of VOCs in exhaled gases may be a promising technique for detecting undiagnosed BE. Relevant studies have been reported but are inadequate, necessitating further research for confirmation.

In 2020, Peters et al. (2020) obtained breath samples from 513 patients and observed no adverse events. Overall, 402 patients were included in the study, with 129 diagnosed with BE, 141 with gastroesophageal reflux disease [including 50 (35.5%) with reflux esophagitis], and 132 in the control group. In the control group, 76 patients (57.6%) had a normal upper gastrointestinal tract or hiatal hernia on endoscopy. The investigators developed and cross-validated a BE prediction model to analyze the VOCs. This eNose could differentiate between patients with and without BE with good diagnostic accuracy [sensitivity, 91%; specificity, 74%; area under the ROC curve (AUC), 0.91] and seemed to be independent of the use of proton pump inhibitors, hiatal hernia, and reflux. Therefore, eNose may be an efficient, well-tolerated, sensitive, and specific screening method, allowing high-risk individuals to be selected for upper gastrointestinal endoscopy.

### eNoses in the diagnosis of colorectal *cancer*

Colorectal cancer (CRC) is the third most frequent malignancy and the foremost contributor to cancer-related mortality. Endoscopic biopsy remains the primary diagnostic method for gastrointestinal tumors. Patients may remain asymptomatic at early or advanced stages of CRC (Chow et al. 1996; Pan and Morrison 2011; Desmond et al. 2019; Park et al. 2020). Because the symptoms of early-stage CRC are nonspecific, the diagnostic rate of endoscopy is suboptimal, and cancer screening is expensive, painful, and unsuitable. Therefore, there is an urgent need for convenient, noninvasive, and low-cost diagnostic methods for the early diagnosis and screening of cancer. Fecal occult blood tests, serum biomarkers, and intestinal barium contrast X-ray angiography are commonly used diagnostic methods for CRC. The fecal occult blood test is currently the most widely used and evaluable method for screening. However, its clinical value is limited because of its high false-positive and false-negative rates. Because of their poor accuracy, serum biomarkers of intestinal tumors, such as carcinoembryonic antigen and cancer antigen 19–9, do not fulfill the expected diagnostic role. While barium contrast X-ray angiography can depict the lesion’s overall location, size, and anatomical relationship with the entire organ, it is radioactive and cumbersome. Therefore, noninvasive biomarkers for the diagnosis of intestinal cancers are needed. The overall declining trend in CRC-related mortality rates is likely due to increased screening, early detection, and improved treatment regimens (Huang et al. 2022). Recently, VOCs have been considered as potential biomarkers of CRC. Therefore, using biomarkers for early detection, diagnosis, and staging is crucial for cancer treatment (Majumdar et al. 1999; Haick et al. 2014; Ogunwobi et al. 2020; Chung et al. 2022).

Peng et al. (2010) first attempted to detect CRC by analyzing the presence of VOCs during exhalation. The results showed that a nanosensor array could differentiate between patients with colon cancer and healthy controls and the breathing conditions in patients with different cancer types, regardless of age, sex, lifestyle, and other confounding factors.

de Meij et al. (2014) used an eNose to assess the odors of disease-specific VOCs in fecal gases to distinguish patients with CRC or advanced adenoma from healthy controls. Stool samples were collected from patients scheduled for elective colonoscopies. The patterns of VOCs in the fecal gases of patients with histopathologically confirmed CRC, those with histopathologically confirmed advanced adenoma, and controls (no anomaly on colonoscopy) were detected using eNose. The CRC and advanced adenoma detection performance was evaluated using ROC curves and calculating the sensitivity and specificity. A total of 157 stool samples (40 from patients with CRC, 60 from those with advanced adenoma, and 57 from healthy controls) were analyzed using eNose. The distribution of stool VOCs in patients with CRC differed significantly from that in the control group [AUC ± 95% confidence interval (CI) 0.92 ± 0.03; *p* < 0.001; sensitivity, 85%; specificity, 87%].

In addition, the VOC profile of patients with advanced adenoma was distinguishable from that of the control group (AUC ± 95% CI 0.79 ± 0.04; *p* < 0.001; sensitivity, 62%; specificity, 86%). These results imply that fecal gas analysis using eNose is a promising novel screening tool for the early detection of advanced neoplasia and CRC.

van Keulen et al. (2020) collected 511 breath samples. Overall, 64 patients were excluded from the study owing to unsatisfactory breath testing (*n* = 51), incomplete colonoscopy (*n* = 8), or colitis (*n* = 5). Patients were classified according to the most advanced lesions, viz. CRC (*n* = 70), advanced adenoma (*n* = 117), non-advanced adenoma (*n* = 117), hyperplastic polyps (*n* = 15), or no anomalies on colonoscopy (*n* = 125). The AUC was 0.76 for CRC and 0.71 for advanced adenoma. The AUCs for CRC and advanced adenoma obtained by blinded validation were 0.74 and 0.61, respectively; the AUCs generated by the CRC and advanced adenoma models were 0.84 (sensitivity, 95%; specificity, 64%) and 0.73 (sensitivity, 79%; specificity, 59%), respectively. This study suggested that exhaled VOCs are potential noninvasive biomarkers for detecting CRC and advanced adenoma. Future studies should include larger samples to improve the potential of VOC analysis for identifying malignant colorectal lesions.

Tyagi et al. (2021) used eNose and gas chromatography–mass spectrometry to differentiate between the CRC group and non-cancer group based on their chemical fingerprints, revealing that eNose had good sensitivity and specificity. Using a neural network classifier, eNose could distinguish between the CRC and non-cancer groups, with an AUC of 0.81, high sensitivity of 91%, and specificity of 55%. Analysis of the CRC and non-cancer groups using a random forest classifier yielded an AUC of 0.80, sensitivity of 82%, and specificity of 55%.

### eNoses in the diagnosis of inflammatory bowel disease

Inflammatory bowel disease (IBD) is an idiopathic, chronic, and nonspecific inflammatory intestinal disorder that can be classified into ulcerative colitis (UC) and Crohn’s disease (CD). The etiology and pathogenesis, which involve genetic susceptibility, environmental triggers (such as diet and lifestyle), and effects on the host microbiome, remain unclear. Bacterial diversity is difficult to study, because only 50% of organisms can be successfully cultured. Although modern genomic technologies can circumvent this problem, they are costly and laborious, making them difficult to adapt to routine clinical use. Among patients with IBD, the endoscopic disease activity level is associated with poor outcomes, and endoscopy remains the most reliable test for evaluating symptomatic patients. However, endoscopy is invasive and imposes physical, psychological, and financial burdens on patients, highlighting the need for noninvasive biomarkers for IBD diagnosis.

Arasaradnam et al. (2013) first used eNose and field asymmetric ion mobility spectrometry (FAIMS) to detect VOCs in the urine of patients with IBD and successfully generated a characteristic chemical fingerprint. They recruited 62 study participants, including 48 patients with IBD (24 with CD and 24 with UC) and 14 healthy controls. The disease activity of the study participants was recorded, and urine samples were collected. The urine samples were analyzed for VOCs using eNose and FAIMS. The eNose data obtained from the experiment were analyzed, and the results showed that eNose could accurately distinguish between patients with IBD and healthy controls, with an accuracy of 0.75% (*p*-value < 0.001).

Tiele et al. (2019) used eNose and a commercial gas chromatography-ion mobility spectrometer to examine the breath samples of patients. The study enrolled 39 participants: 14 were diagnosed with CD, 16 with UC, and 9 served as controls. Both methods could distinguish patients with IBD from controls, with eNose technology having an AUC ± 95% CI of 0.81 ± (0.66–0.96), sensitivity of 67%, and specificity of 89%. In addition, this method could differentiate UC from CD, with eNose technology having an AUC ± 95% of 0.88 ± (0.77–0.98), sensitivity of 71%, and specificity of 88%.

## Fundamental challenges and limitations

eNose may not be sensitive enough to detect certain VOCs, which may restrict the diagnostic accuracy for certain diseases. Due to the possible differences in the types and concentrations of VOCs produced in different diseases, it may be difficult for eNose to distinguish between similar odor patterns, diminishing its sensitivity. As multiple diseases may produce similar patterns of VOCs, eNoses may have difficulty in accurately distinguishing between these diseases. In addition, individual differences (such as age, sex, lifestyle habits, etc.) may also affect the production and release of VOCs, further reducing the specificity of eNose. Since odor is subjective, and different people may perceive and describe the same smell differently, the standardization and accuracy of these devices is beset by challenges. The semiconductor gas sensor currently in use cannot fully meet the above-mentioned requirements, especially in terms of selectivity and stability. Therefore, devising new semiconductor sensor processing technology and the improvement of sensor performance are important future development directions.

Another obvious limitation of VOC detection in biological samples is interference factors such as humidity, temperature and other compounds (water, salt, protein, etc.). Filters, temperature compensation, packaging, and sealing technologies are mainly used to overcome humidity. Other interference factors can be eliminated by establishing a strict quality control system, including regular calibration of sensors, verification of detection methods, and evaluation of test results, to ensure the accuracy and reliability of the test results.

Furthermore, due to the complexity and high specialization of eNose technology, its equipment (viz. sensor materials, manufacturing processes, signal processing, pattern recognition, etc.) and research and development costs are relatively high, limiting promotion and application in some fields. These technical limitations may restrict diagnostic performance. However, with advancements in biochips, microelectronics, computers, material science, etc., eNose technology is expected to continue to overcome the existing technical limitations and gain wider applications in more fields.

## Conclusions

Rapid and accurate gastrointestinal disease diagnosis is crucial for correct and timely treatment. Delayed treatment and the use of inappropriate acid-suppressive drugs worsen disease conditions and result in high morbidity and mortality rates for tumors, and increase the cost and burden of medical care. Current methods for detecting digestive diseases have certain limitations and applying potential VOCs in the clinical diagnosis remains challenging. First, potential confounding factors that influence VOCs, such as human metabolic activities and external environmental changes, must be overcome. Second, techniques related to the capture of VOCs must be standardized, and the analytical techniques used to extract potential biomarkers from complex datasets must be simplified. In addition, multi-center clinical cohort studies with large sample sizes are needed to validate and reduce the variability between the results of various independent studies. The disadvantage of eNose is the lack of absolute calibration and measurement information, which limits its use in clinical practice.

eNose technology explores how biological olfactory functions can be imitated. Research involves science and technology in various specific application fields, including materials, precision manufacturing processes, multisensor fusion, computers, and applied mathematics. Therefore, it is important to study the theoretical significance and application prospects of the technological aspects of eNose. eNose has garnered attention as a noninvasive medical testing and clinical diagnostic technology. eNose can facilitate early diagnosis and screening of certain diseases and rapid detection of microbial infections by virtue of being portable and facilitating real-time, online, and in situ analysis. This has important clinical value. With the emergence of new sensitive materials (e.g., biomaterials), the invention and application of new sensing principles and technologies (e.g., photoelectric technology), and in-depth research on information processing, eNose are expected to find extensive and intensive applications in medical diagnostics.

## Data Availability

No datasets were generated or analysed during the current study.

## Abbreviations

AUC: Area under the curve
CD: Crohn’s disease
CI: Confidence interval
CRC: Colorectal cancer
DFA: Discriminant factor analysis
eNOSE: Electronic nose
FAIMS: Field asymmetric ion mobility spectrometry
GC: Gastric cancer
IBD: Inflammatory bowel disease
OLGIM: Operative Link on Gastric Intestinal Metaplasia
ROC: Receiver operating characteristic
UC: Ulcerative colitis
VOC: Volatile organic compound
